# Gene duplication and the environmental regulation of physiology and development

**DOI:** 10.1002/ece3.1099

**Published:** 2014-05-06

**Authors:** David C Gibbs, Kathleen Donohue

**Affiliations:** Department of Biology, Duke UniversityBox 90338, Durham, North Carolina, 27708

**Keywords:** Developmental timing, genetic redundancy, niche breadth, phenology, phenotypic plasticity

## Abstract

When different life stages have different environmental tolerances, development needs to be regulated so that each life stage experiences environmental conditions that are suitable for it, if fitness is to be maintained. Restricting the timing of developmental transitions to occur under specific combinations of environmental conditions is therefore adaptively important. However, impeding development can itself incur demographic and fitness costs. How do organisms regulate development and physiological processes so that they occur under the broadest range of permissive conditions? Gene duplication offers one solution: Multiple genes contribute to the same downstream process, but do so under distinct combinations of environmental conditions. We present a simple model to examine how environmental sensitivities of genes and how gene duplication influence the distribution of environmental conditions under which an end process will proceed. The model shows that the duplication of genes that retain their downstream function but diverge in environmental sensitivities can allow an end process to proceed under more than one distinct combination of environmental conditions. The outcomes depend on how upstream genes regulate downstream components, which genes in the pathway have diversified in their sensitivities, and the structure of the pathway.

## Introduction

In environments that vary over time, which include any location with seasons, the environmental regulation of development and physiology can be a major determinant of fitness. Because different life stages frequently have different tolerances to external environmental conditions, the perception of environmental cues and the regulation of the timing of developmental transitions in response to those cues can effectively match each life stage to its appropriate environment. For instance, seeds or eggs can be highly resistant to dessication, while young seedlings and hatchlings can be highly vulnerable to it. Therefore, the seasonal timing of germination or hatching can be under strong natural selection (Fernandez-Quintanilla et al. 1986; Jones et al. 1997; Purrington and Schmitt 1998; Seiwa 1998; Visser and Holleman 2001; Donohue 2002; Shimono and Kudo 2003; Donohue et al. 2005; Castro 2006; Benard and Toft 2007; van Asch et al. 2007; Weekley et al. 2007; Watanuki et al. 2009; Moriyama and Numata 2011; reviewed in Donohue et al. 2010). The ability to restrict such developmental transitions to occur only under specific combinations of permissive environmental conditions can determine life or death.

Conversely, over-specialization of developmental cues can be detrimental. If triggers for developmental transitions are highly restrictive, then development may not proceed when those conditions are not met. This can in itself impose low to zero fitness via perpetual developmental arrest, or if it is a temporary restriction, it can impose a demographic cost associated with delayed growth and deferred reproduction (Cohen 1976; Bull and Shine 1979; Tuljapurkar 1990). Such environmental restrictions at any life stage have the potential to narrow the ecological niche of an organism and potentially restrict its geographic range (reviewed in Donohue et al. 2010).

Environmental sensitivity to multiple environmental cues has the potential to impose highly restrictive conditions for development to proceed, potentially imposing restrictions to development even under suitable conditions. For instance, suitable conditions may include warm, wet conditions, or cool, dry conditions, but not warm, dry conditions. If development were to be inhibited under all warm conditions, then the organism would incur the cost of impeded development even under a subset of favorable conditions (e.g., warm, wet). How can organisms regulate their development or physiological processes to occur over the widest possible range of permissive conditions, while still restricting development to specific combinations of suitable environmental factors?

Redundancy of genetic pathways offers one solution, namely when more than one environmentally sensitive pathway contributes to the same downstream physiological or developmental process. Each pathway may promote the downstream process under its own set of restrictive environmental conditions. Combined over all such pathways, the end process can proceed under multiple highly specific combinations of environmental conditions. This can effect precise environmental regulation of development without imposing restrictions to development under permissive conditions.

Gene duplication may be an especially efficient form of such “redundancy,” because upon duplication, a duplicated gene copy already contributes to the same pathway and end process as its paralog. That is, a common pathway can be regulated by more than one gene copy, but those duplicated genes can have divergent environmental sensitivities. If the environmentally dependent function of duplicated copies diverges, then the downstream process could occur under more than one distinct combination of environmental conditions.

While duplicated gene copies frequently regulate distinct developmental or physiological processes, important examples are known in which duplicated genes contribute to the same physiological or developmental process. In some cases, this conservation of function is thought to be imposed by an adaptive advantage of increased dosage of the end product of the pathway (Haberer et al. 2004; Li et al. 2005; Ganko et al. 2007; Edger and Pires 2009; Qian et al. 2010). In other cases, the duplicated gene copies have been shown to contribute to the same end process, but under different environmental conditions. For example, the phytochrome gene family is derived from a series of gene duplications (Sharrock and Quail 1989; Clack et al. 1994; Mathews and Sharrock 1997), and duplicated phytochromes have diverged in both gene expression regulation and in coding sequence. Over the past decade, it has been shown that multiple phytochromes contribute to the same developmental process of seed germination, but the contribution of each phytochrome depends on the temperature during seed maturation and after dispersal as well as chilling and light (Shinomura et al. 1994; Poppe and Schafer 1997; Shinomura 1997; Hennig et al. 2001, 2002; Koornneef et al. 2002; Heschel et al. 2007, 2008; Donohue et al. 2008; Holdsworth et al. 2008). This differential contribution of the phytochromes could be caused by differences in environment-dependent gene expression (Quail 1994; Somers and Quail 1995; Goosey et al. 1997; Sharrock and Clack 2002) and differences in environmental sensitivities of their gene products (Kendrick and Spruit 1977; Shinomura et al. 1996; Braslavsky et al. 1997; Clough and Vierstra 1997; Elich and Chory 1997; Casal and Sanchez 1998; Eichenberg et al. 2000). Downstream of the phytochromes, the family of duplicated gibberellin oxidase genes regulates the conversion of inactive to bioactive gibberellins, which stimulate germination (Ritchie and Gilroy 1998; Yamaguchi et al. 1998; Yamaguchi and Kamiya 2000; Holdsworth et al. 2008; Yamaguchi 2008). As with the phytochromes, the contribution of specific gibberellin oxidase genes to GA metabolism and thereby germination depends on environmental conditions, such as chilling (Yamauchi et al. 2004; Yamaguchi 2008). Thus, the phytochrome-mediated germination pathway involves duplicated genes with distinct environmental regulation of their activity, both upstream in the signal transduction pathway and downstream at the point of metabolism of a gene product that is a major stimulant of the final developmental process of germination. The prevalence of this manner of involvement of duplicated genes in the environmental regulation of development and physiology is not known, but other examples in diverse taxa have found associations between gene duplication and environmental responsiveness (Goldman et al. 2006; Liu and Adams 2007; Hanada et al. 2008; Zou et al. 2009).

Here, we present a simple two-locus model of an environmentally regulated developmental/physiological process, to examine how the environmental sensitivities of genes in the pathway and how gene duplication influence the distribution of environmental conditions under which an end process will proceed. In particular, we are interested in conditions that result in more than one peak of physiological activity across the distribution of possible environmental conditions and the conditions that result in the restriction of the process to occur around those peaks. In this manner, we investigate conditions that enable the precise environmental regulation of an end process but that allow it to proceed under more than one distinct combination of suitable environmental conditions.

With this model, we begin by examining conditions that produce the most precise restriction of the final process around a specific combination of environmental conditions, when there is no diversification of duplicated gene copies. We first examine the relative effects of environmental regulation of upstream versus downstream genes and how combinations of environmental optima of genes in the pathway translate to the environmental probability distribution under which the end process occurs. Specifically, we compare outcomes when upstream, downstream, or both genes are environmentally sensitive. Next, we consider the effects of divergence in the environmental sensitivities of duplicated gene copies of both upstream and downstream genes. In particular, we compare outcomes when upstream, downstream, or both genes are divergent in environmental sensitivities. Finally, we show that the structure of the pathway with duplicated genes influences the environmental probability distribution of the final process.

## Model Format

**Figure 1:**
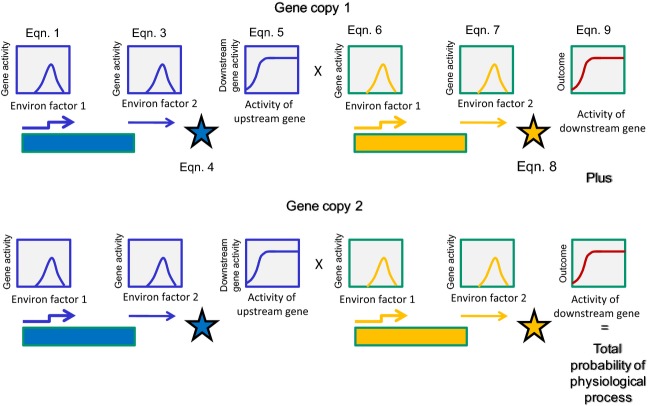
Schematic of the model of environmental regulation of a physiological or developmental process with duplicated genes. Duplicated pathways contribute to the same physiological or developmental outcome. Each gene copy can exhibit sensitivity to two environmental factors, given by a function with an intermediate optimum. This environmental sensitivity can be caused by environment-dependent gene expression or environment-dependent activity of the gene product. The expression of the downstream gene is a sigmoidal function of the amount of active gene product from the upstream gene. Its expression and activity are further contingent on the same two environmental factors as those that regulate the upstream gene activity. The final physiological process is a sigmoidal function of the sum of the amount of active gene product of the downstream gene from both gene copies. Equations referred to in the upper panel are in Appendix.

The model investigates how a physiological or developmental process can be restricted to proceed under specific combinations of two environmental factors and how gene duplication within that pathway alters the range of environmental conditions under which that process can proceed. This simple genetic pathway model has two genes: an upstream gene and a downstream gene. Each upstream and downstream gene has two duplicated copies that can be independently influenced by two environmental factors. The upstream gene's activity is a function only of the two environmental factors, while the downstream gene's activity is a function of both the environmental factors and the upstream gene's activity. The final physiological outcome is a function of the downstream gene's activity. Finally, the model is structured so that we can compare the effects on the physiological outcome when each downstream gene copy is regulated independently by a single upstream copy versus when each downstream gene copy is regulated by the combined (or “pooled”) activities of both upstream copies. Schematic depictions of these pathways are shown in Fig. 1, and equations are provided in the Appendix.

In this model, the two genes exhibit activity as a function of their sensitivities to two environmental factors, e1 and e2. Environment-dependent gene activities are modeled as a function with an intermediate optimum, with activity that diminishes to a value of zero above and below the optimum environmental value, at a rate given by *α* (for environmental factor 1) or *β* (for environmental factor 2). Many enzymes have activities that reflect functions with intermediate optima, with kinetic activity that increases with temperature, for example, until a maximum, above which protein denaturation occurs (Peterson et al. 2007 and references therein). Similar activity functions have been shown in response to pH (Millat et al. 2013 and references therein). Other environmental functions, such as responses to photoperiod, may be monotonic such that the probability of flowering increases with increasing photoperiod length up to a threshold (Welch et al. 2003). The model here can accommodate such functions by changing values of *α*_0_ relative to *α*_1_ and *β*_0_ relative to *β*_1_ (see Appendix for further explanation).

The total activity (maximum of 1) of the upstream gene is the product of the probability of the gene activity in response to environmental factor 1 and the probability of the gene activity in response to environmental factor 2. This multiplicative probability of gene activity can be interpreted, for example, as the amount of active gene product produced as a function of environment-dependent gene expression in response to the environmental factors, environment-dependent gene product activity, or a combination of both.

The activity of the downstream gene is first a sigmoidal function of the amount of gene activity (active product) of the upstream gene, bounded by zero and 1 as the maximum. The steepness of this function and point of inflection is given by the parameters *δ*_0_ and *δ*_1_, respectively. Transcriptional regulation of developmental genes is frequently modeled as sigmoidal functions of the concentration of its activator (Nahmad et al. 2008 and references therein), and sigmoidal response functions can result from regulators that exhibit environmental sensitivities with intermediate optima (Millat et al. 2013). Additionally, downstream gene activity is a function of the two environmental factors, as above. Its total activity (active product) is therefore a function of upstream gene activity multiplied by the probability of the downstream gene activity under environmental factor 1 and the probability of the downstream gene activity under environmental factor 2.

The final physiological outcome is a sigmoidal function of the total gene activity (active product) of the downstream gene, and the steepness of this function and point of inflection is given by the parameters *γ*_0_ and *γ*_1_, respectively. A sigmoidal function of the final physiological response reflects basic enzyme kinetics of a saturation of reaction rate as a function of enzyme concentration (Kacser and Burns 1981), as well as commonly observed responses to hormones (e.g., Barua et al. 2012 for germination response to GA concentration). This final outcome is quantitative, in the sense that it indicates the probability of the outcome occurring. This interpretation is similar to developmental threshold approaches that model the accumulation of developmental units, with the developmental transition occurring once a threshold number of developmental units have been attained (Welch et al. 2003), except that this model concerns continuous functions rather than thresholds.

We present results when the upstream and/or downstream gene copies have duplicated and diversified in environmental sensitivities, focusing on diversification in the environmental optima for gene activity. As a final comparison, we present results in which each of the two downstream gene copies is regulated independently by a given upstream gene copy; in other words, the activity of upstream gene copy 1 regulates the activity of downstream gene copy 1, and the activity of upstream gene copy 2 regulates the activity of downstream gene copy 2. Such independent regulation is known to occur, for example, in the case of different phytochromes regulating different copies of downstream gibberellin oxidase genes (Yamauchi et al. 2004; Mitchum et al. 2006). In contrast, both downstream gene copies may be regulated as a function of the total amount of upstream gene activity, summed over both gene copies of the upstream gene; that is, each downstream gene's activity is a function of the activity of upstream gene copy 1 plus the activity of upstream gene copy 2.

The model output presented here gives the developmental/physiological outcome value as a function of the two environmental factors' values, in the form of a two-dimensional surface heat map. Each environmental factor is scaled from 1 to 20, and the modeling parameters are chosen so that the physiological outcome values are between 0 and 1 (to represent the probability that the final physiological response occurs within a certain combination of the two environmental factors' values).

## Results

### No diversification of gene copies

To examine conditions that produce the most precise restriction of the final process around a specific combination of environmental conditions, we first compare the relative effects of environmental regulation of upstream versus downstream genes with no diversification of environmental sensitivities of duplicated gene copies. Specifically, we assume duplicated gene copies have identical sensitivities, and we compare outcomes when upstream, downstream, or both genes are environmentally sensitive. Here, upstream or downstream gene activity is set to be maximal across the full range of environmental values (no sensitivity) or to exhibit an optimal level of activity as a function of the two environmental factors, as described above.

**Figure 2:**
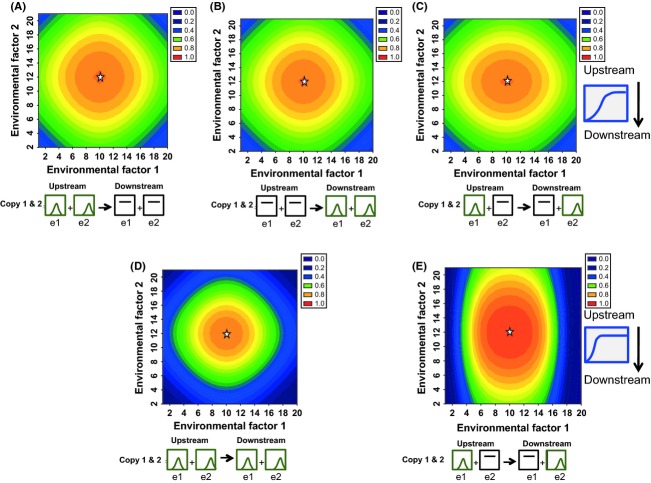
Upstream versus downstream environmental regulation, with no diversification of gene copies. Heat map of the probability that a physiological function will occur as a function of environmental factor 1 (*x*-axis) and environmental factor 2 (*y*-axis). White stars indicate the environmental conditions that correspond to peak activity of the environmentally sensitive genes. Graphics below each panel illustrate in a qualitative manner the environmental sensitivity functions (for environmental factor 1 and environmental factor 2, e1 and e2, respectively) of the upstream and downstream genes (left of arrow and right of arrow, respectively). Flat lines indicate that the gene has maximal activity across the full range of the environmental factor (i.e., it does not exhibit sensitivity to that factor). (A) Only the upstream gene is environmentally sensitive, with peak activity when environmental factor 1 = 10 and environmental factor 2 = 12 (e1_opt_ = 10 and e2_opt_ = 12). (B) Only the downstream gene is environmentally sensitive, with peak activity when e1_opt_ = 10 and e2_opt_ = 12. (C) The upstream gene is sensitive to environmental factor 1 (e1_opt_ = 10), and the downstream gene is sensitive to environmental factor 2 (e2_opt_ = 12). (D) Both upstream and downstream genes exhibit identical environmental sensitivities (e1_opt_ = 10 and e2_opt_ = 12 for upstream and downstream genes). (E) Environmental sensitivity functions are as in C, but the sigmoidal function that describes how the activity of the upstream gene regulates the downstream gene (icons to right) is steeper than in C. For A–D, *α* = 0.01; *β* = 0.01 when genes exhibit environmental sensitivity (when lack of sensitivity in the form of a flat function is present, *α* and/or *β* = 0); *δ*_0_, *δ*_1_ = 1, 2; *γ*_0_, *γ*_1_ = 1,2. For e, parameters are the same except *δ*_0_, *δ*_1_ = 5, 7.

The outcomes of environmental sensitivity of upstream versus downstream genes are comparable when the sigmoidal functions of upstream and downstream gene functions on downstream processes are equal (*δ*_1_ = *γ*_1_ and *δ*_2_ = *γ*_2_). Specifically, the range of environmental conditions under which the physiological/developmental outcome occurs is similar regardless of whether the upstream or downstream gene exhibits environmental sensitivity, provided the function describing the effect of the upstream gene's activity on downstream gene expression (*δ*) is the same as the function of the effect of the downstream gene on the final outcome (*γ*). With these conditions, the physiological outcome proceeds with the greatest frequency under conditions that are closest to the environmental optima of gene activity (Fig. 2A and B). Comparable outcomes also result when one gene (either upstream or downstream) exhibits sensitivity to both factors (Fig. 2A and B), compared to when upstream genes are sensitive to one factor, while the downstream gene is sensitive to the other (Fig. 2C). More precise environmental regulation occurs when both upstream and downstream genes exhibit identical environmental sensitivities, such that the physiological outcome is more tightly restricted to conditions closer to the environmental optima for gene activity (Fig. 2D).

In contrast, when the sigmoidal functions of upstream and downstream genes differ (*δ*_1_ ≠ *γ*_1_ and *δ*_2_ ≠ *γ*_2_), the position within the pathway at which environmental sensitivity occurs does influence the conditions under which the final process occurs (Fig. 2E). Specifically, when the sigmoidal function is steeper, the environmental regulation is more precise, such that the physiological process occurs within a tighter range around the environmental optimum. For example, in Fig. 2E, the upstream gene is sensitive to environmental factor 1 (*x*-axis) and has a steeper sigmoidal function regulating the activity of the downstream gene than it does in Fig. 2C; the physiological outcome proceeds within a narrower range of factor 1 in Fig. 2E than it does in Fig. 2C.

**Figure 3:**
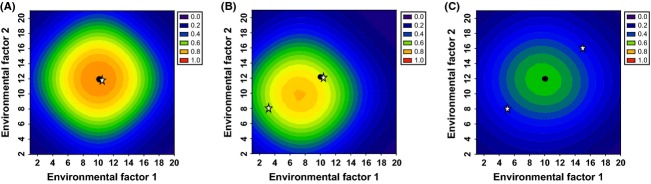
Suboptimal sensitivities of one gene can be compensated for by suboptimal sensitivities for another gene, but reduce total occurrence. Heat map of the probability that a physiological function will occur as a function of environmental factor 1 (*x*-axis) and environmental factor 2 (*y*-axis). The adaptively optimal condition for the physiological process to occur is indicated by the black circle. In all cases, the adaptively optimal condition occurs when environmental factor 1 = 10 and environmental factor 2 = 12. Stars indicate the conditions for maximal gene activity for the upstream and downstream genes. (A) Both the upstream and downstream genes have peak activity under the adaptively optimal conditions (stars have been jittered for visibility), namely when e1_opt_ = 10 and e2_opt_ = 12. (B) The upstream gene has peak activity under optimal conditions (for upstream gene, e1_opt_ = 10 and e2_opt_ = 12; white star jittered), but the downstream gene has peak activity under suboptimal conditions (for downstream gene, e1_opt_ = 5 and e2_opt_ = 8). The peak physiological activity does not occur under the adaptively optimal condition. (C) The downstream gene has peak activity under suboptimal conditions, as in B (for downstream gene, e1_opt_ = 5 and e2_opt_ = 8), and the peak activity of the upstream gene also has peak activity under suboptimal conditions in a compensatory manner (for upstream gene, e1_opt_ = 15 and e2_opt_ = 16), so that the peak physiological activity now occurs under adaptively optimal conditions. All other parameters are as in Fig. 2D.

We next consider how the environmental optima for gene activity reflect adaptively optimal conditions for the developmental/physiological process to occur. In Fig. 3, the adaptively optimal environmental condition for the physiological process to proceed is shown by the black circle. In Fig. 3A, both genes have peak activities under conditions that are also adaptive optima. In Fig. 3B, however, the downstream gene has an optimal activity that does not correspond to the adaptive optimum, and the peak physiological process occurs with the highest probability under conditions that are not adaptively optimal. However, when the environmental optima of the upstream gene are changed in a complementary manner, so that they also no longer match the adaptive optima (Fig. 3C), then the physiological process can occur with the highest probability under the adaptively optimal combination of environmental conditions. Therefore, a maladaptive environmental optimum of one gene in the pathway can be compensated for by suboptimal environmental sensitivity of the other gene in the pathway. This suggests that environmental sensitivities of genes within a pathway can contribute to epistasis for fitness, such that the optimal environmental sensitivity function of one gene depends on the environmental sensitivity function of other genes in the pathway. Importantly however, the total probability of the physiological process occurring under adaptively optimal conditions is less with compensatory changes in the other gene than without them (Fig. 3B vs C); that is, compensation by another gene can shift the peak probability back over the optimal condition, but the probability of the physiological event occurring under those conditions diminishes. This result suggests a potential cost even of compensatory changes in environmental sensitivity, in the form of decreasing the likelihood that development will continue at all under those conditions. Alternatively, if the probability refers to the probability per unit time, this result could indicate that the process proceeds more slowly with compensatory changes of environmental optima for gene activities than without them.

### With diversification of gene copies

**Figure 4:**
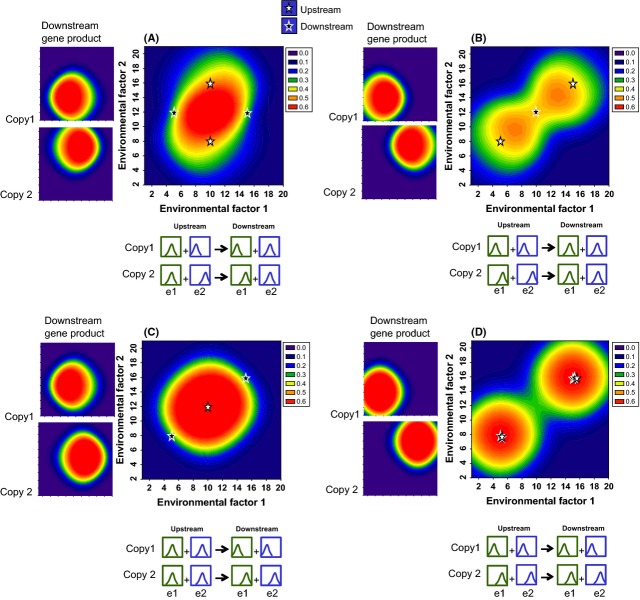
Multiple peaks of physiological activity with diversification of gene copies. Heat map of the probability that a physiological function will occur as a function of environmental factor 1 (*x*-axis) and environmental factor 2 (*y*-axis). White stars and black stars indicate the environmental conditions that correspond to peak activity of the environmentally sensitive upstream and downstream genes, respectively. Graphics below each panel illustrate in a qualitative manner the environmental sensitivity functions, as in Fig. 2. Side panels show the downstream gene product activity generated by each gene copy, and axes are the same as in the main panels. Optimum value for environmental factor 1 and environmental factor 2 = e1_opt_ and e2_opt_, respectively. When gene copies are not divergent, e1_opt_ = 10 and e2_opt_ = 12. When gene copies are divergent, e1_opt_ = 5 and e2_opt_ = 8 for copy 1, and e1_opt_ = 15 and e2_opt_ = 16 for copy 2. (A) The upstream duplicated gene copies are divergent for sensitivity to environmental factor 2, and the downstream gene copies are divergent for sensitivity to environmental factor 1. Copy1: upstream gene e1_opt_ = 10, e2_opt_ = 8, downstream gene e1_opt_ = 5, e2_opt_ = 12; copy 2 upstream gene e1_opt_ = 10, e2_opt_ = 16, downstream gene e1_opt_ = 15, e2_opt_ = 12. (B) The upstream gene copies are divergent for sensitivities to both environmental factors, and the downstream gene copies are not divergent for sensitivity to either environmental factor. Copy1: upstream gene e1_opt_ = 5, e2_opt_ = 8, downstream gene e1_opt_ = 10, e2_opt_ = 12; copy 2 upstream gene e1_opt_ = 15, e2_opt_ = 16, downstream gene e1_opt_ = 10, e2_opt_ = 12. (C) The downstream gene copies are divergent for sensitivities to both environmental factors, and the upstream gene copies are not divergent for sensitivity to either environmental factor. Copy1: upstream gene e1_opt_ = 10, e2_opt_ = 12, downstream gene e1_opt_ = 5, e2_opt_ = 8; copy 2 upstream gene e1_opt_ = 10, e2_opt_ = 12, downstream gene e1_opt_ = 15, e2_opt_ = 16. (D) Both upstream and downstream gene copies have identically divergent sensitivity to both environmental factors. Copy1: upstream gene e1 = 5, e2 = 8, downstream gene e1 = 5, e2 = 8; copy 2 upstream gene e1 = 15, e2 = 16, downstream gene e1 = 15, e2 = 16. For a-d, *α* = 0.01; *β* = 0.01; *δ*_0_, *δ*_1_= 5, 7; *γ*_0_, *γ*_1_= 1,2. Note that the upstream sigmoidal function is steeper than the downstream sigmoidal function in all panels (*δ* > *γ*). Note also that the *z*-axis differs from that in Figs 2 and 3.

As shown above, with the addition of environmental sensitivities of genes in a pathway, the range of conditions under which a physiological or developmental process will proceed becomes increasingly more restrictive (Fig. 2C versus D). This may be considered to be an improvement in the precision of physiological regulation when environmental optima correspond to adaptive optima. However, a given value of an environmental factor may be adverse when in combination with some values of another environmental factor, but favorable when in combination with other values of that factor. Increasing the environmental restrictions may increase the precision of the environmental regulation of a physiological process, but it cannot result in the physiological process proceeding under more than one favorable combination of environmental factors. In contrast, gene duplication can (Fig. 4B and D).

To test how divergence in the environmental sensitivities of duplicated gene copies influence the range of environments under which a physiological/developmental outcome can occur, we compare outcomes when upstream, downstream, or both genes are divergent in environmental sensitivities. With divergence of environmental sensitivities of duplicated gene copies, the range of environmental conditions under which the physiological process proceeds is expanded, and this expansion is restricted to more than one distinct peak of activity under some conditions (Fig. 4). It should be noted that such diversification can also reduce the total probability of the physiological process occurring (Fig. S1). Moreover, the environmental conditions that promote the peak physiological activity may not correspond with the environmental conditions that produce the peak activities of the individual genes, as explained next.

First, the environmental conditions that correspond to the peak physiological activity are closer to the environmental optimum for the upstream gene when it has a steeper sigmoidal function of downstream regulation than the downstream gene (when *δ* > *γ*); this is apparent in Fig. 4A–C from the observation that the peaks of the heat maps tend to match the environmental optima for the upstream gene (white stars) more closely than those of the downstream genes (black stars). Moreover, when upstream gene copies are divergent and downstream gene copies are not, distinct peaks of physiological activity occur (Fig. 4B); in contrast, when downstream gene copies are divergent, but upstream gene copies are not, a single broad peak of physiological activity occurs (Fig. 4C). When the upstream gene copies are divergent for sensitivity to environmental factor 1, and downstream gene copies are divergent for environmental factor 2, the peak of physiological activity is more restricted with respect to environmental factor 1 than environmental factor 2 (Fig. 4A). Thus, the sigmoidal function describing how upstream pathway components regulate downstream components can influence the range and precision of physiological activity as a function of the environment.

Second, the degree to which the peak physiological activity matches the peak sensitivity of individual genes varies according to how similarly upstream and downstream gene copies have diverged. The most precise matching occurs when upstream and downstream gene copies are identically divergent with respect to their environmental sensitivities (Fig. 4D).

**Figure 5:**
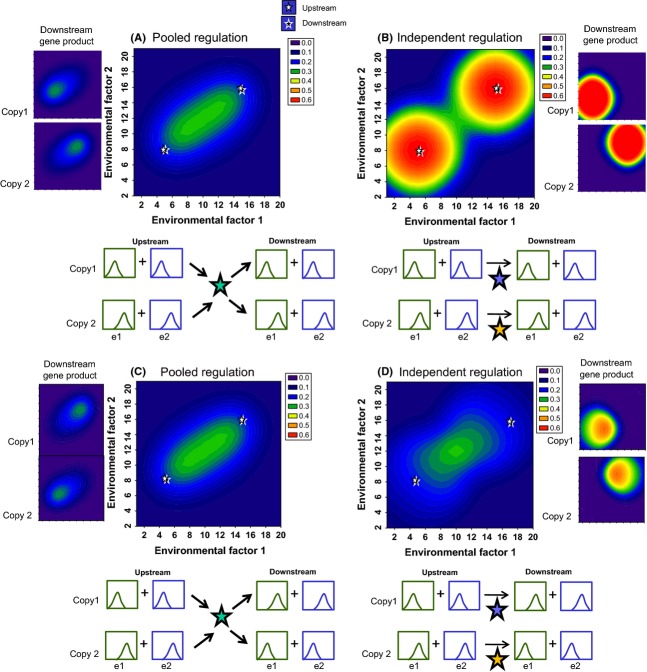
Pooled versus independent regulation of downstream gene expression. Heat map of the probability that a physiological function will occur as a function of environmental factor 1 (*x*-axis) and environmental factor 2 (*y*-axis). White stars and black stars indicate the environmental conditions that correspond to peak activity of the environmentally sensitive upstream and downstream genes, respectively (stars are jittered so that both are visible). Side panels show the downstream gene product activity generated by each gene copy; axes are the same as in the main panels. Graphics below each panel illustrate in a qualitative manner the environmental sensitivity functions, as in Fig. 2. (A) Pooled regulation: The activity of each downstream gene is a function of the total activity of both upstream genes (green star in lower panel). (B) Independent regulation: The activity of downstream gene copy 1 is a function of the activity of upstream gene copy 1 (blue star in lower panel), and the activity of downstream gene copy 2 is a function of the activity of upstream gene copy 2 (yellow star in lower panel). That is, gene duplication of both copies results in two independent pathways that contribute to the physiological process. For A and B, copy 1 upstream and downstream genes have e1_opt_ = 5 and e2_opt_ = 8; copy 2 upstream and downstream genes have e1_opt_ = 15 and e2_opt_ = 16. (C) Pooled regulation and d) independent regulation, as in A and B, but the upstream and downstream genes do not have the same environmental optima. For upstream gene copy 1 and downstream gene copy 2, e1_opt_ = 5 and e2_opt_ = 8. For upstream gene copy 2 and downstream gene copy 1, e1_opt_ = 15 and e2_opt_ = 16. Other model parameters are the same as in Fig. 4D. Note that the *z*-axis differs from that in Figs 2 and 3, but are the same as in Fig. 4.

Finally, when upstream and downstream gene copies are identically divergent, independent regulation of downstream gene copies by specific upstream gene copies results in more precise matching of the peak physiological activity to peak sensitivity of individual genes than when the activity of the downstream gene is a function of the pooled activity of the upstream genes (Fig. 5A versus B). Regulation of downstream genes by pooled upstream gene activity also results in a lower total probability of the physiological process. Thus, when environmental sensitivities of genes correspond to adaptive optima for a physiological or developmental process to occur, the diversification of upstream gene copies appears to enhance the benefit of downstream duplication, because subsequent downstream divergence would increase the precision of regulation of the final outcome around specific combinations of environmental conditions.

When upstream and downstream gene copies are not identically divergent, however, independent regulation of downstream gene copies by specific upstream genes does not result in more precise matching of physiological activity with peak activity of individual genes (Fig. 5C and D). In fact, the pooled regulation of downstream genes (Fig. 5D) can actually increase the probability that the end process occurs under conditions that are optimal for gene activity when upstream and downstream genes are not identically divergent in their environmental sensitivities.

## Discussion

This model of a simple genetic pathway with gene duplication shows that gene duplication of genes that retain their downstream function but diverge in environmental sensitivities can restrict a physiological process to occur under specific environmental conditions, but allow it to proceed under more than one distinct combination of environmental conditions. Thus, gene duplication can impose precise environmentally cued development or physiology, but enable development/physiology to proceed under a broader range of potentially optimal conditions than pathways without duplication. Its ability to match environmental conditions of peak physiological activity to that which is adaptively optimal, however, depends on how the upstream components regulate downstream components, which genes in the pathway have diversified in their sensitivities, and the structure of the pathway itself.

First, the efficiency whereby upstream pathway components regulate downstream components can influence the distribution of environmental conditions under which the final outcome occurs; the steeper the function of gene regulation (the sigmoidal function here), the more restricted the process is to occur near the environmental optima of the regulating gene. When these regulatory functions differ across the pathway, the environmental distribution of peak physiological activity will depend on both the environmental optima of each gene and the steepness of the regulatory function of that gene.

Second, the most precise restriction of physiological activity around discrete combinations of environmental factors occurs when both upstream and downstream genes are identically environmentally sensitive. Divergent environmental sensitivities may produce similar peaks of physiological activity as identically divergent sensitivities, such that changes in environmental sensitivities of one gene can be compensated for by changes in other genes in the pathway in a manner that preserves the environmental distribution of physiological activity. This suggests an interesting source of epistasis for fitness when the environmental distribution of physiological activity is under selection. This is likely to be the case for seasonal phenology, which determines the seasonal environment under which developmental transitions occur, such as hatch-out/germination, metamorphosis, transitions to reproduction/flowering, etc., and which has been shown to be a major determinant of fitness (Bradshaw and Holzapfel 2008; reviewed in Donohue et al. 2010).

Although suboptimal environmental sensitivities can compensate for each other to produce peak physiological activities that correspond to adaptively optimal conditions, the total probability of the physiological outcome under those conditions is lower. Whether this compensation is actually more adaptive than not compensating depends on how strongly fitness is reduced by the process occurring under nonoptimal conditions compared to the cost of reducing the probability it occurs even under optimal conditions. If those optimal conditions are stable and persistent, however, then the physiological or developmental process may be able to proceed to comparable levels, even though it may take longer.

Future theoretical work that examines the fitness consequences of both the relative and absolute environmental distribution of physiological activity would be informative for exploring these dynamics as a potential source of epistasis for fitness. Such studies should examine both fitness consequences of a process occurring under suboptimal environmental conditions and the demographic cost of delaying the process even under favorable conditions. Empirical studies that investigate correlational selection on combinations of (biochemical or gene expression/activity) phenotypes or combinations of alleles that differ in environmental sensitivities could test such hypotheses directly. Such experiments offer a particularly rich area for future study because they could quantify both the fitness costs of proceeding with development under suboptimal conditions and the demographic costs of postponing development until conditions are closer to the optimum.

Finally, the pathway structure itself influenced the environmental distribution of a physiological or developmental process. When environmental sensitivities are identical in genes within a given pathway, pathways that maintain independent regulation of downstream gene copies by specific upstream gene copies produce more precise environmental restriction of the physiological process than pathways in which downstream genes are regulated by pooled products of the upstream genes. Such independent regulation of downstream duplicated genes by upstream duplicated genes is known to occur (e.g., Yamauchi et al. 2004; Mitchum et al. 2006). As such, when precise environmental regulation is adaptive, upstream gene duplication could create a situation in which downstream duplication and subsequent specialization of interactions among upstream and downstream gene copies is adaptive. Before paired upstream and downstream gene copies acquire similar environmental sensitivities, however, the evolution of independent regulation of downstream genes by specific upstream gene copies may entail an overall reduction in the probability that the final process occurs under conditions that promote peak gene activity. It would therefore be interesting to investigate whether paired environmental sensitivities of genes in a pathway evolve prior to independent regulation, or vice versa.

The aim of this model was to predict the range of environmental conditions under which a physiological or developmental process will occur, but it can also provide insight into other processes of adaptive significance. For example, the compensatory phenomenon we observed, whereby a change in the environmental sensitivity in one locus can be compensated for by a change in the sensitivity of a second locus to restore the conductions under which peak activity occurs, has some similarity with “Developmental System Drift” in which different gene expression profiles in a gene network can result in the same phenotype (Abouheif et al. 1997; True and Haag 2001; Nahmad et al. 2008). Differences in expression profiles that conserve a phenotype can evolve through genetic drift, selection on gene expression—either direct or indirectly via selection on alternative phenotypes controlled by the same gene—or a combination of both (Nahmad et al. 2008). Exploring the adaptive consequences of such compensatory systems of environmental regulation and their evolutionary dynamics could provide insight into the more general phenomena of developmental system drift and parallel evolution.

In addition, this model predicts the probability of a process occurring under a range of environmental conditions. The topography of this probability surface reflects how responsive a phenotype is to environmental perturbation (Debat and David 2001; Meiklejohn and Hartl 2002; Espinosa-Soto et al. 2011), with a flat surface indicating strong phenotypic robustness to environmental perturbations and a more textured surface indicating greater responsiveness of phenotypes to environmental perturbation. Studies of phenotypic robustness have shown that responsiveness to nongenetic perturbations can facilitate the evolution of phenotypic novelty via genetic assimilation (Espinosa-Soto et al. 2011). Our study suggests that diversification of environmental sensitivities in duplicated genes can affect the evenness of the landscape of environmental responsiveness of developmental phenotypes.

The environmental regulation of development has been shown to be one of the most important mechanisms whereby diverse organisms respond to environmental change (Parmesan and Yohe 2003; Chuine et al. 2004; Menzel et al. 2006; Parmesan 2006; Bradshaw and Holzapfel 2008), whether through climate change or dispersal and range expansion. The range of environmental conditions under which key developmental transitions or physiological functions occur has the potential to be a strong determinant of the breadth of ecological environments organisms inhabit. Environmental cuing of development can, on the one hand, enable habitat selection of specific life stages by matching life stages to the seasonal conditions they can withstand; on the other hand, it can restrict the range of environmental conditions under which organisms can complete their life cycle altogether. Understanding the genetic basis of such environmental regulation of physiology and development could provide information necessary to predict organismal responses to environmental variation experienced across their geographic range and environmental changes in the future and suggest mechanisms whereby they may adapt to such variation.

## Appendix 1: Model formulation

### Upstream Gene

The upstream gene activity is regulated by two environmental factors given by the functions in Eqns A1–A3. Equation A1 describes the gene activity when environmental conditions are below the optimum, and Eqn. A2 describes gene activity when conditions are above the optimum. The first subscript indicates whether the environmental value is the optimum (o) or actual (a) value, and the second subscript indicates whether the gene is upstream (u) or downstream (d). Therefore, e1_*o,u*_ is the upstream gene's optimum value for environmental factor 1, and e1_*a,u*_ is the actual value for environmental factor 1. The activity of the upstream gene, contingent on environmental factor 1, is

(A1)

(A2)

When the actual value is below the optimum value, the upstream gene's activity approaches zero, as it deviates from the optimal value, at a rate denoted by *α*_0,u_, which is also indicative of the variance around the optimum (Eqn. A1). When the actual value is above the optimum, the upstream gene's activity controlled by environmental factor 1 approaches zero at a rate denoted by *α*_1,u_ (Eqn. A2). This formulation allows for asymmetric responses above and below the optimum. All following functions of environmental responses can also be formulated to be asymmetric. All results presented here are from symmetric environmental responses, where *α*_0,u_ = *α*_1,u_ = *α*_u_, so only one function is presented for each environmental response from this point forward.

The activity of the upstream gene, contingent on environmental factor 2, is

(A3)

where e2_o,u_ is the upstream gene's optimal value for environmental factor 2 and e2_a,u_ is the actual value of environmental factor 2 experienced by the upstream gene. Like *α*_u_, *β*_u_ is the rate at which the upstream gene activity controlled by environmental factor 2, A_u_(e2), approaches zero as conditions deviate from the optimal value. In sum, the sensitivity to both environmental variables has optimal values, e1_o_ and e2_o_, which approach zero at a rate denoted by *α* and *β,* respectively.

The upstream gene's total activity, A_u_, is the gene activity controlled by environmental factor 1 multiplied by the gene activity controlled by environmental factor 2 (Eqn. A4).

(A4)

A multiplicative, as opposed to additive, probability was used in order to investigate scenarios in which the process can proceed only if both environmental conditions are present. This criterion reflects the commonly observed phenomenon that a combination of environmental conditions is required for a developmental process to progress (for example, the regulation of flowering as a function of vernalization combined with permissive temperature, or the regulation of germination as a function of dormancy-breaking treatments followed by permissive germination conditions). Additive functions might occur in nature, such that one promoter allows a process to occur under one condition and another promoter allows the process to occur under another condition. We focus here on the combinatorial probability, however, because we are interested in scenarios that restrict events to occur under combinations of environments, in a manner that reflects commonly observed developmental regulation.

### Downstream Gene

The level of downstream gene expression (E_d_) produced by the upstream gene's activity (A_u_) is given by the following sigmoidal function (Eqn. A5).

(A5)

where *δ*_0_ determines the steepness of the sigmoidal curve and *δ*_1_ determines the point of inflection of the curve.

The downstream gene activity is also a function of two environmental factors, in a similar manner as the upstream gene. The downstream gene's activity, contingent on environmental factor 1, is

(A6)

where e1_o,d_ is the downstream gene's optimal value for environmental factor 1 and e1_a,d_ is the actual value for environmental factor 1 experienced by the downstream gene. As in Eqn. A1, *α*_d_ is the rate at which the downstream gene's activity, A_d_(e1), approaches zero as it deviates from the optimal value of environmental factor 1.

Likewise, the downstream gene's activity, contingent on environmental factor 2, is

(A7)

where e2_o,d_ is the downstream gene's optimal value for environmental factor 2, e2_a,d_ is the actual value of environmental factor 2 experienced by the downstream gene, and *β*_d_ is the rate at which the downstream gene's activity approaches zero as it deviates from the optimal value of environmental factor 2.

The total activity of the downstream gene, (A_d_), is equal to the downstream expression controlled by upstream activity, E_d_(A_u_)*,* multiplied by the combined downstream gene activities controlled by environmental factors 1 and 2 (Eqn. A8).

(A8)

### Physiological Outcome

The physiological outcome (P) is a sigmoidal function of the total activity of the downstream gene, A_d_ (Eqn. A9):

(A9)

where *γ*_0_ determines the steepness of the sigmoidal curve and *γ*_1_ determines the point of inflection of the curve.

## Supporting Information

**Figure S1.** Gene duplication can result in multiple peaks of developmental/physiological activity in response to environmental conditions.
