# Supplementary material for: Gene duplication and the environmental regulation of physiology and development
Source: Ecol Evol. 2014 May 6;4(11):2202–16. doi: 10.1002/ece3.1099 (PMC4201434; doi:10.1002/ece3.1099)
Supplement: Supplementary file 1 — Figure S1. Gene duplication can result in multiple peaks of developmental/physiological activity in response to environmental conditions. [file ece30004-2202-sd1.docx]

**SUPPLEMENTAL INFORMATION**

Figure S1. Gene duplication can result in multiple peaks of developmental/physiological activity in response to environmental conditions. Direct comparison of the probability (given as heat map) that a physiological process will occur as a function of environmental factor 1 (x-axis) and environmental factor 2 (y-axis) for diversified and non-diversified gene copies, with identical axes. White stars and black stars indicate the environmental conditions that correspond to peak activity of the environmentally sensitive upstream and downstream genes respectively (jittered in b and c). Graphics below each panel illustrate in a qualitative manner the environmental sensitivity functions, as in Figure 2. a) Only one gene is sensitive to a given environmental factor. Parameters are identical to those in Fig. 2c. b) Both upstream and downstream genes are identically environmentally sensitive. Parameters are identical to those in Fig. 2d. c) Upstream and downstream genes of copy 1 are identically environmentally sensitive, and upstream and downstream genes of copy 2 are identically environmentally sensitive but divergent from copy 1 genes. Parameters are identical to those in Fig. 4d.
